# TiO_2_ Nanowire Networks Prepared by Titanium Corrosion and Their Application to Bendable Dye-Sensitized Solar Cells

**DOI:** 10.3390/nano7100315

**Published:** 2017-10-12

**Authors:** Saera Jin, Eunhye Shin, Jongin Hong

**Affiliations:** Department of Chemistry, Chung-Ang University, 84 Heukseok-ro, Dongjak-gu, Seoul 06974, Korea; saera0907@gmail.com (S.J.); swh0904@gmail.com (E.S.)

**Keywords:** TiO_2_, wet corrosion, dye-sensitized solar cells

## Abstract

TiO_2_ nanowire networks were prepared, using the corrosion of Ti foils in alkaline (potassium hydroxide, KOH) solution at different temperatures, and then a further ion-exchange process. The prepared nanostructures were characterized by field emission scanning electron microscopy, Raman spectroscopy, and X-ray photoelectron spectroscopy. The wet corroded foils were utilized as the photoanodes of bendable dye-sensitized solar cells (DSSCs), which exhibited a power conversion efficiency of 1.11% under back illumination.

## 1. Introduction

The dye-sensitized solar cell (DSSC, or Grätzel cell) was first presented in 1991 [1], and has since been considered a low-cost alternative to conventional silicon solar cells, and a promising construction element for building-integrated photovoltaics (BIPV). This is due to its transparency and diverse colors [2]. Recently, cost-effective (Ti and stainless steel) metal foils have been proposed for the fabrication of bendable DSSCs for flat and curved building skins instead of plastic materials. This is primarily because of the lack of limitations with respect to high-temperature processing [3,4,5,6]. In particular, Ti substrates decreased the series resistance of DSSCs, and thus allowed for a better fill factor (FF) and power conversion efficiency, as compared with F-doped SnO_2_ (FTO)-conducting glasses [5,6]. The DSSC consists of a dye-sensitized porous photoanode, a redox electrolyte, and a platinized counter electrode. Photoanodes are generally prepared using TiO_2_ nanoparticles with a size of 5–20 nm. However, such mesoporous nanoparticle films are hampered by the limited electron transport caused by particle-to-particle hopping and charge recombination at interfaces [7,8]. With the aim of improving the DSSC performance, one-dimensional (1D) TiO_2_ nanostructures, such as nanotubes, nanorods, and nanowires, have been synthesized by various methods including anodization [9,10,11], electrospinning [12] and hydrothermal alkali treatment of titania nanoparticles [13,14]. It is suggested that one-dimensional (1D) nanostructures offer better transport pathways for photogenerated electrons than nanoparticles because of their longer carrier diffusion lengths [8,15]. Recently, 1D titania arrays have been directly prepared on a Ti foil via the anodic oxidation of Ti in different electrolytes containing fluoride ions [16], and have been used as the photoanode of DSSCs [9,11,17]. The 1D titania arrays could also be detached and transferred onto the FTO glass for the fabrication of DSSCs [17]. However, it is not easy to reliably produce such nanostructures over a large area, since the anodization process is sensitive to electrochemical reaction conditions. Direct oxidation of Ti foils can also produce nanorods or nanowires directly grown on Ti substrates, but they require a long growth time [18] or high temperatures [19]. In this study, we report a facile approach for fabricating TiO_2_ nanowire networks on Ti foil using a Ti corrosion reaction in KOH aqueous solutions at different temperatures, followed by a further ion-exchange process. We also investigate the efficacy of the TiO_2_ nanowire networks as photoanodes for bendable DSSCs.

## 2. Results and Discussion

**Figure 1:**
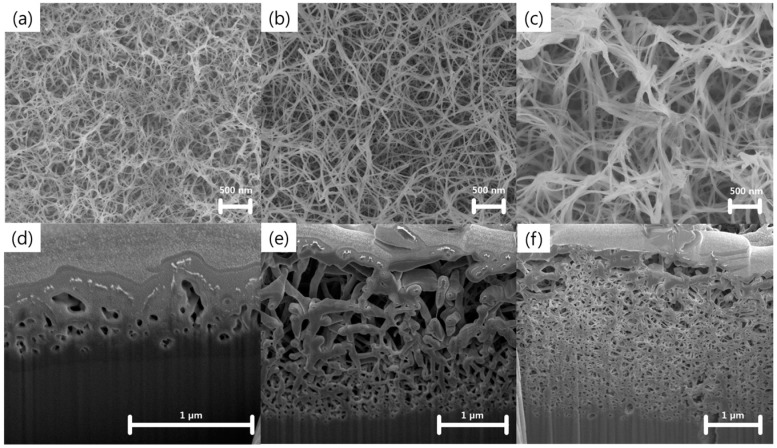
Field emission scanning electron microscopy (FE-SEM) images of the surface and cross-section of wet-corroded Ti foil at various temperatures in 5 M KOH aqueous solution: (**a**,**d**) 20 °C, (**b**,**e**) 50 °C, and (**c**,**f**) 80 °C.

**Figure 2:**
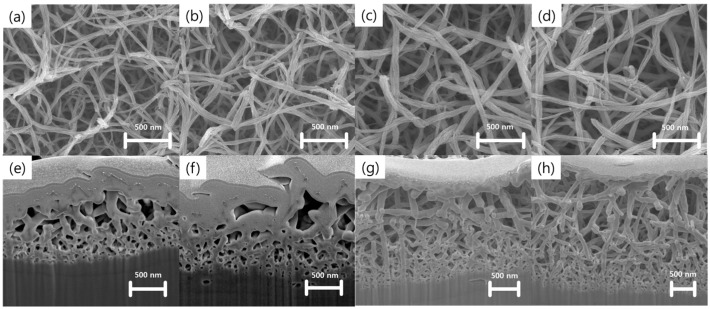
FE-SEM images of the surface and cross-section of wet-corroded Ti foil at 50 °C in 5 M KOH aqueous solution for various time periods: (**a**,**e**) 6 h, (**b**,**f**) 12 h, (**c**,**g**) 24 h, and (**d**,**h**) 48 h.

Figure 1 shows field emission scanning electron microscopy (FE-SEM) images of the nanostructures prepared using Ti wet corrosion in 5 M KOH aqueous solution at different temperatures. Porous nanowire networks were formed on the Ti surface at all corrosion temperatures. Details of the chemical reactions occurring between Ti and alkaline solution can be found elsewhere [20,21]. Ti reacts with hydroxide ions through hydration reactions, which results in hydrated TiO_2_. Meanwhile, hydrated TiO_2_ can be also dissolved as negatively charged hydrates by hydroxyl attack. Notably, a higher corrosion temperature yielded thicker nanowires, networks that were more aggregated, and a deeper corrosion depth. Figure 2 shows FE-SEM images of nanowire networks formed in 5 M KOH at 50 °C, and compares various corrosion time periods. Interestingly, compact nanowire networks were formed near the metal/oxide interface, whereas porous networks were produced near the surface. This result might be related to the local concentration gradient of hydroxide ions. The average thicknesses of the nanostructures formed for corrosion times of 6, 12, 24, and 48 h were 1.4 ± 0.1, 1.6 ± 0.2, 2.6 ± 0.2, and 3.3 ± 0.1 μm, respectively. 

**Figure 3:**
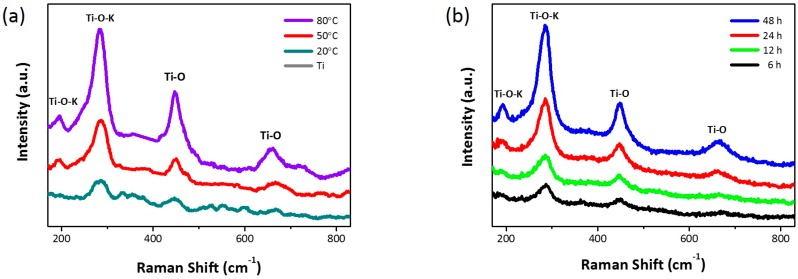
Raman spectra of Ti foils corroded (**a**) in 5 M KOH aqueous solution for 24 h at different temperatures and (**b**) in 5 M KOH aqueous solution at 50 °C for different time periods.

As reported previously, the resulting nanostructures could be K-incorporated TiO_2_ nanowires containing K–Ti–O bonds [20,21,22,23,24]. Figure 3 shows the Raman scattering spectra of all of the wet-corroded samples under 531 nm excitation to identify Ti–O bonds of the anatase and rutile phases and K–Ti–O bonds of potassium titanates. Anatase has six Raman active modes (i.e., 144 cm^−1^ (E_g_), 197 cm^−1^ (E_g_), 399 cm^−1^ (B_1g_), 513 cm^−1^ (A_1g_), and 639 cm^−1^ (E_g_)) and rutile has four Raman active modes (i.e., 143 cm^−1^ (B_1g_), 447 cm^−1^ (E_g_), 612 cm^−1^ (A_1g_), and 826 cm^−1^ (B_2g_)) [25]. All samples showed typical Raman peaks originating from the anatase and rutile phases of TiO_2_ (i.e., 197 cm^−1^, 448 cm^−1^, 640 cm^−1^_,_ and 826 cm^−1^). Interestingly, prominent Raman peaks resulting from the potassium-doped TiO_2_ (K-doped TiO_2_; i.e., 285 cm^−1^ and 660 cm^−1^) were also observed [26,27]. As the reaction temperature increased, all of the peaks became more intense and sharper, as a result of which the crystallinity of the nanostructures improved. Similarly, the corrosion time also had an influence on the degree of crystallinity. The X-ray diffraction (XRD) patterns of the wet-corroded Ti foils were also obtained (Figure S1 in Supplementary Materials). The intensity of the crystalline Ti peaks decreased with the increase in the reaction temperature. Unfortunately, prominent crystalline TiO_2_ peaks were not observed using XRD.

**Figure 4:**
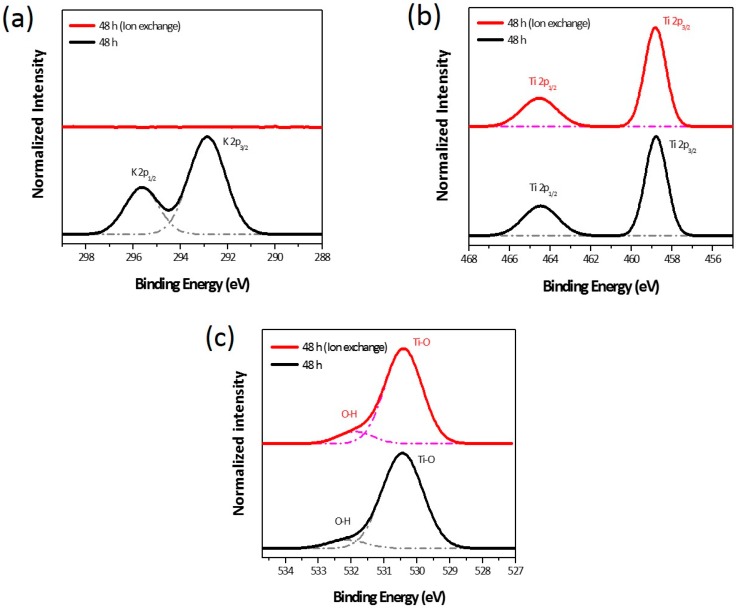
Normalized intensity of the XPS narrow scan spectra of (**a**) K 2p, (**b**) Ti 2p, and (**c**) O 1s levels. The dotted lines below the XPS spectra represent the Lorentzian-fitted curves.

The chemical identity of the wet-corroded samples was confirmed by X-ray photoelectron spectroscopy (XPS). To replace K^+^ with H^+^, the wet-corroded Ti foil was immersed in HCl solution and the efficacy of the ion-exchange (hereafter referred to as “ion-exchange” and abbreviated as “IE”) was investigated as well. According to the survey XPS scans, the samples contained K, Ti, and O; no other elements were detected, except for carbon (Figure S2 in Supplementary Materials). Figure 4 shows the X-ray photoelectron narrow scan spectra of the K 2p, Ti 2p, and O 1s levels in the nanostructures prepared at 50 °C for 48 h. The observed binding energies of the K 2p, Ti 2p, and O 1s levels are summarized in Table 1. As can be seen from Figure 4a, the binding energy of the K 2p_3/2_ level before IE treatment was in fairly good agreement with that of the K-incorporated titanates reported in the literature. However, the K 2p peak disappeared completely after the IE treatment. As shown in Figure 4b, the Ti 2p doublet peaks of Ti 2p_1/2_ and Ti 2p_3/2_ were observed at 464.5 eV and 458.8 eV, respectively, and this is ascribed to the Ti–Ti bond. In Figure 4c, the binding energy of the O 1s level corresponds mainly to the Ti–O bond (bulk O^2−^) in TiO_2_ (530.0 eV and 530.5 eV for anatase and rutile, respectively). The small peak at the higher binding energy was a result of OH groups belonging to hydroxyl groups and adsorbed H_2_O, and its intensity became slightly higher after the IE treatment. On the basis of the observed binding energies, the as-received nanostructures were concluded to be K-doped TiO_2_. Unfortunately, the K-doped TiO_2_ exhibited p-type characteristics [24], and thus the removal of potassium dopant was crucial in order to use the nanostructures as the DSSC photoanode. It should be noted that the IE process was highly effective in removing potassium from the nanostructures without affecting the Ti–Ti and Ti–O bonds.

**Figure 5:**
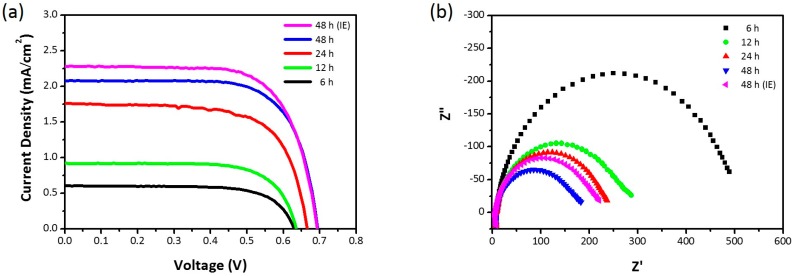
(**a**) J-V characteristics and (**b**) Nyquist plots of DSSCs.

The fabricated nanowire networks were sensitized with N719 dye on the Ti foil and then used as the photoanode of a DSSC. However, because the substrate was metallic, the DSSC was required to be illuminated from the Pt counter electrode side (i.e., back illumination). The main drawback of this configuration relates to the transmission losses due to the Pt-based catalyst and the *I*^−^/*I*_3_^−^ liquid electrolyte. Figure 5a shows the current density-voltage (J-V) characteristics of the DSSCs under back illumination. Table 2 summarizes the photovoltaic parameters. The photovoltaic performance improved with an increase in the reaction time: open-circuit voltage (*V_oc_*) increased from 0.63 V to 0.69 V; photocurrent density (*J_sc_*) increased from 0.60 mA/cm^2^ to 2.08 mA/cm^2^; and power conversion efficiency (*η*) increased from 0.27% to 1.03%. Notably, the IE treatment resulted in better photovoltaic performance: *η* increased from 1.03% to 1.11%. Electrochemical impedance spectroscopy (EIS) can offer valuable insights into interfacial charge-transfer processes of DSSCs. Figure 5b shows the Nyquist plots of the DSSCs under back illumination with applied open-circuit voltage. The semicircle in the intermediate frequency region reflects the charge-transfer resistance at the TiO_2_/photosensitizer/electrolyte interface. As the photoanode thickness increased, the charge-transfer resistance decreased; thus, this coincides with the resultant DSSC performance.

## 3. Material and Methods 

### 3.1. Three-Dimensional TiO_2_ Nanowire Networks

A pure titanium foil (Ti > 99.5%, Nilaco Co., Tokyo, Japan) with a thickness of 1 mm was used as the starting material for wet corrosion. TiO_2_ nanostructures could be prepared through a Ti corrosion reaction in KOH aqueous solution [20]. A Ti substrate 15 mm × 30 mm in size was polished with a SiC sheet (No. 1000) and subsequently cleaned by ultrasonication in acetone, isopropanol, and deionized (DI) water. The cleaned substrate was immersed in 5 M KOH (95%) for 24 h at different temperatures (20, 50, and 80 °C). The wet-corroded Ti substrate was thoroughly rinsed with DI water. The 3D morphology of the TiO_2_ nanostructures was investigated by field emission scanning electron microscopy (FE-SEM, S-4800, Hitachi, Tokyo, Japan). A Focused Ion Beam (FIB, Thermo Fisher Scientific, Waltham, MA, USA) was used to prepare the cross-sectional samples. Micro-Raman spectroscopy was performed in a back-scattering geometry by using a laser operating at a wavelength of approximately 531 nm and with a spectral resolution of 1.4 cm^−1^ (FEX, NOST, Seongnam, Korea). The Raman signals were detected using a charge-coupled-device (CCD) camera (iDus DV401A, Andor, Concord, MA, USA). The XRD patterns were collected on a D/max250/PC (Rigaku, Tokyo, Japan) using Cu radiation at 40 kV and 200 mA at room temperature. X-ray photoelectron spectroscopy (XPS) was performed with the K-Alpha XPS system (Thermo Fischer Scientific, Waltham, MA, USA) using a monochromated Al Kα X-ray source with an energy of 1486.6 eV. The spectra of Ti 2p and O 1s energy levels were calibrated with respect to the C 1s peak of the adventitious carbon on the sample surface at 285.0 eV.

### 3.2. DSSCs

The wet-corroded foil was immersed in 0.1 M HCl aqueous solution for 24 h at room temperature (RT) to replace K^+^ with H^+^, after which it was rinsed with deionized (DI) water and dried under N_2_ flow. The titanium tetrachloride (TiCl_4_) treatment was performed by soaking the foil in 0.04 M TiCl_4_ aqueous solution at 75 °C for 30 min. It was then rinsed with DI water and sintered at 500 °C for 30 min. The foil was exposed to O_2_ plasma and then immersed in 0.1 M HNO_3_ solution for 30 min to facilitate dye adsorption. The final foil was immersed in a 0.5 mM N719 (Solaronix) ethanol solution for 12 h. A Pt counter electrode was prepared on fluorine-doped SnO_2_ (FTO)-coated conducting glass (TEC 8, Pilkington; thickness: 2.2 mm, sheet resistance: 8 Ω/sq) by spin-coating of 0.04 M chloroplatinic acid (H_2_PtCl_6_) solution and post-annealing at 400 °C for 1 h. Both the dye-sensitized foil and the Pt counter electrode were sealed with a 25-μm-thick layer of Surlyn (Solaronix, Aubonne, Switzerland). An iodide based redox electrolyte (Iodolyte AN-50, Solaronix, Aubonne, Switzerland) was injected into the cell. The photovoltaic characteristics of the cell were measured using a solar cell I–V measurement system (K3000 LAB, McScience Inc., Suwon, Korea) under air mass 1.5 (AM 1.5) global, one-sun illumination (100 mW/cm^2^). The effective area of the fabricated solar cell was 1 cm × 0.7 cm. The open-circuit voltage (*V_oc_*), photocurrent density (*J_sc_*), fill factor (*FF*), and power conversion efficiency (*η*) were recorded simultaneously. EIS experiments were performed using a frequency response analyzer (Solartron 1260, AMETEK. Inc., Berwyn, PA, USA). A sinusoidal potential perturbation with an amplitude of 10 mV was applied over a frequency range from 100 kHz to 0.1 Hz.

## 4. Conclusions

TiO_2_ nanowire networks were easily prepared with Ti corrosion in strong basic solutions at different temperatures and then a further IE process. Importantly, the prepared nanostructures on Ti foils were utilized as the photoanodes of bendable DSSCs, and consequently, the DSSCs exhibited a power conversion efficiency of 1.11%, even under back illumination. Our work towards further developments (e.g., fabrication optimization and transfer of the TiO_2_ nanowire networks to various substrates [11,17,28] for front illumination) will be explored and published in due course. 

## Figures and Tables

**Table 1:** Binding energies of K 2p, Ti 2p, and O 1s levels in X-ray photoelectron spectroscopy (XPS) fitting. IE: ion exchange.

(Unit: eV)	K 2p		Ti 2p		O 1s	
	2p_1/2_	2p_3/2_	2p_1/2_	2p_3/2_	O–H	Ti–O
48 h	295.6	292.9	464.5	458.8	532.1	530.4
48 h (IE)	-	-	464.5	458.8	531.9	530.4

**Table 2:** Photovoltaic characteristics of wet-corroded Ti foil dye-sensitized solar cells (DSSCs) at 50 °C for different corrosion time periods. IE: ion exchange; FF: fill factor; *V_oc_*: open-circuit voltage; *η*: power conversion efficiency; *J_sc_*: photocurrent density.

Wet Corrosion Time (h)	*V_oc_* (V)	*J_sc_* (mA/cm^2^)	*FF* (%)	*η* (%)
6	0.63	0.60	72.0	0.27
12	0.64	0.92	71.7	0.42
24	0.67	1.76	68.3	0.80
48	0.69	2.08	71.5	1.03
48 (IE)	0.69	2.28	70.1	1.11

## Supplementary Materials

The following are available online at http://www.mdpi.com/2079-4991/7/10/315/s1, Figure S1: XRD patterns of the wet-corroded Ti foil samples at various temperatures in 5 M KOH aqueous solution and normal Ti foil, Figure S2: Survey XPS spectrum of wet-corroded Ti foil sample at corrosion temperature of 50 °C and corrosion time of 48 h.
