# Supplementary material for: TiO2 Nanowire Networks Prepared by Titanium Corrosion and Their Application to Bendable Dye-Sensitized Solar Cells
Source: Nanomaterials (Basel). 2017 Oct 12;7(10):315. doi: 10.3390/nano7100315 (PMC5666480; doi:10.3390/nano7100315)
Supplement: Supplementary file 1 [file nanomaterials-07-00315-s001.pdf]

TiO_2 Nanowire Networks Prepared by Titanium Corrosion and Their Application to Bendable Dye-Sensitized Solar Cells

Saera Jin †, Eunhye Shin † and Jongin Hong *

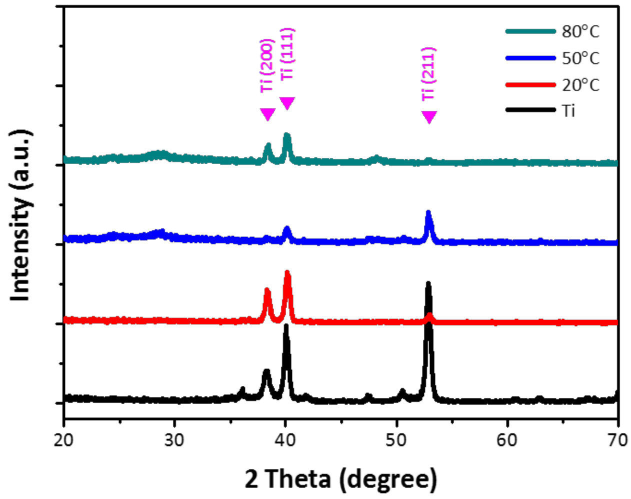

Figure S1. XRD patterns of the wet-corroded Ti foil samples at various temperatures in 5 M KOH aqueous solution and normal Ti foil.

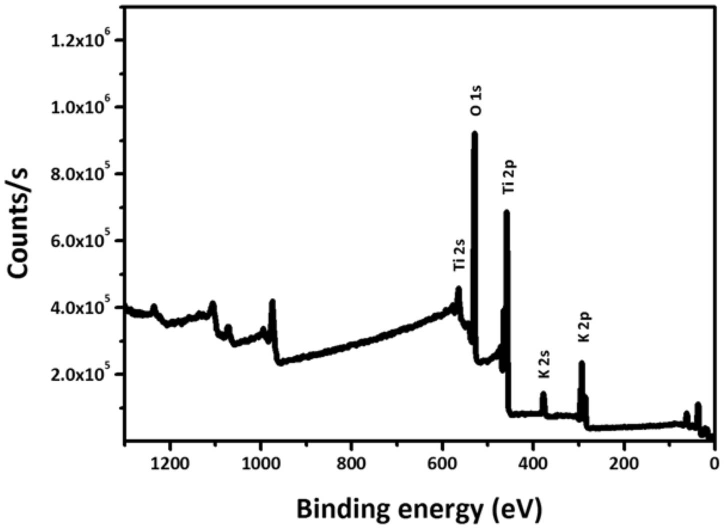

Figure S2. Survey XPS spectrum of wet-corroded Ti foil sample at corrosion temperature of 50°C and corrosion time of 48h.
